# Supplementary material for: Impact of gestational low-protein intake on embryonic kidney microRNA expression and in nephron progenitor cells of the male fetus
Source: PLoS One. 2021 Feb 5;16(2):e0246289. doi: 10.1371/journal.pone.0246289 (PMC7864410; doi:10.1371/journal.pone.0246289)
Supplement: S1 File — (DOCX) [file pone.0246289.s001.docx]

**Supporting Information:** The manuscript data were available in: [https://www.ncbi.nlm.nih.gov/sra/PRJNA694197](https://www.ncbi.nlm.nih.gov/sra/PRJNA694197)

in [https://bv.fapesp.br/pt/pesquisador/671860/leticia-de-barros-sene/](https://bv.fapesp.br/pt/pesquisador/671860/leticia-de-barros-sene/)
[https://repositorio.unesp.br/handle/11449/148594](https://repositorio.unesp.br/handle/11449/148594)
