# Supplementary material for: Impact of gestational low-protein intake on embryonic kidney microRNA expression and in nephron progenitor cells of the male fetus
Source: PLoS One. 2021 Feb 5;16(2):e0246289. doi: 10.1371/journal.pone.0246289 (PMC7864410; doi:10.1371/journal.pone.0246289)
Supplement: Attachment — Submitted filename: Response to Reviewers v.08-12-20.docx [file pone.0246289.s003.docx]

Response to Reviewers

PONE-D-20-15837

 The gestational low-protein intake impact in microRNA expression of the kidney progenitor cells in male offspring fetuses

Dear Editor:

We are attaching the re-revised version and response to the Reviewers of the Manuscript PONE-D-20-15837. The gestational low-protein intake impact in microRNA expression of the kidney progenitor cells in male offspring fetuses submitted by Sene et al. resubmitted as a full paper. We want to beware of the errors during the responses to the pertinent comments emanating by Reviewer 2 during the first evaluation of this manuscript. There is no justification for the carelessness that occurred. However, all occurred without bad faith and can be partially justified by the inexperience of the Author and the inattentive reading of the senior Author, who at the same time answered other criticisms from reviewers of other manuscripts sent for publication. I have read and have abided by the statement of ethical standards for documents submitted to the Plos One and the additional information that all authors have approved the final article. This manuscript has not been submitted or published in any other journal. At first, thank you very much for the reviewer's comments, suggestions, and criticisms. Practically all sections of the manuscript were rewritten entirely, experiments are redone, the number of experiments has been increased, and many reviewer suggestions were now included in that new version. The grammatical and typographical errors have been corrected. The Introduction, Material, and Method and Discussion sections of the manuscript were revised and completely rewritten to include reviewers' suggestions and comments.

Hopefully, this edited manuscript version could now be better considered for publication by this prestigious Journal.

Sincerely yours,

**Patricia Aline Boer, Ph.D**

**Internal Medicine Department, School of Medicine,**

**Campinas State University, Campinas, SP, Brazil.**

Reviewer 2 comments to the Author

Reviewer #2: The authors have significantly improved the manuscript, both scientifically and language-wise. However, several flaws remain to be addressed. Specific comments follow below:

 1. The authors were not as careful as they should be in their reply, copying and pasting their first response to reviewer 1 as their first response to reviewer 2, including the term ”reviewer 1”.

 2. It is unclear which experiments were redone; please specify them.

 3. It is unclear which experiments had their numbers increased; please specify them.

Response from the authors:

REF.: PONE-D-20-15837

**R.: 1, 2, and 3 questions.**

We want to thank Reviewer 2 very much for the spending time and careful reading and to beware of the errors during the responses to the pertinent comments emanating during the first evaluation of this manuscript. There is no justification for the carelessness that occurred. However, all occurred without bad faith and can be partially justified by the inexperience of the Author and the inattentive reading of the senior Author, who at the same time answered other criticisms from reviewers of other manuscripts sent for publication. As suggested, the document was submitted for revision by a native English speaker. Among the errors that occurred during the response to Reviewer 2, the authors stated that they remade and carried out new experiments. This gross error for which we apologize was due, as stated above, to the simultaneous response of papers being reviewed simultaneously. Embarrassed, we apologize again. We have much appreciated your comments and suggestions. Practically, all manuscript sections were entirely rewritten, and many reviewer suggestions were included in that new version. The Introduction, Material, and Method and Discussion sections of the manuscript were revised and completely rewritten to include the suggestions and comments of reviewer 2.

 4. It is true that the language quality improved. However, the amount of sentence structure/clarity problems and grammatical mistakes remains above an acceptable level, both in the manuscript and in reply to the reviewers. Please have the manuscript go through a native English speaker.

**R.: 4 comment**

As suggested, the document was submitted for revision by a native English speaker.

 5. The authors have appropriately addressed my previous major comments 1 and 2, however such a point is unclear in the current manuscript. Please add this information to the revised version of the manuscript.

**Response to comment 5:** The changes were included in Figures 1 and 2 legends to read:

**Figure 1.** Expression of miRNAs in the metanephros from the 17th day LP fetus compared to their expression level in the control group. Reference genes U6 and U87, protein complexes composed of small nuclear RNAs (snRNAs), were used to normalize each miRNA expression. The authors established a cutoff point variation of 1.3 (upwards) or 0.65 (downwards), and data are expressed as fold change (mean ± SD, n = 4) concerning the control group. * p≤0.05: statistical significance versus NP.

**Figure 2.** Expression of mRNA estimated by SyBR green RT-qPCR of metanephros from the 17th day LP fetus. The expression was normalized with GAPDH. The authors established a cutoff point variation of 1.3 (upwards) or 0.65 (downwards), and data are expressed as fold change (mean ± SD, n = 4) concerning the control group. * p≤0.05: statistical significance versus NP.

 6. The modified versions of sentences 3.1 and 3.7 (re: previous major comment 3) are understandable but remain inappropriately written.

**R.: 6 comment**

Sentence 3.1 was changed to read:

Authors demonstrated that gestational low-protein (LP) intake offspring presents a lower birth weight, reduced nephron numbers and renal salt excretion, arterial hypertension, and renal failure development compared to regular protein (NP) intake rats in adulthood. The current study evaluated the different miRNAs and kidney predicted target gene expression in gestational 17-days LP (DG-17) fetal metanephros seeking to elucidate some of the molecular pathways involved in the proliferation and differentiation of renal embryonic/fetal cell.

Sentence 3.7 was changed to read:

By the way, the exhaustion of the remaining CM progenitor cell endowment, in turn, predispose reduced nephron numbers, arterial hypertension development, and renal disorders in adult age (Figure 10). In the current study, confirming Boivin et al. (2015), we may state that increased CM β-catenin might disrupt UB ramifications and nephrogenesis. Have been previously known that growth factor glial-derived neurotrophic factor (GDNF) genes encoding, a crucial regulator of UB outgrowth, acts via c-Ret tyrosine kinase receptor and Gfra1 co-receptor (Brodbeck et al., 2004; Davis et al., 2015). In the 17-DG LP offspring, a significant increase in c-Ret receptor coding mRNA would theoretically lead to a rise in UB ramifications. However, in the present study, the GDNF expression was unchanged, being plausible to suppose that, despite the increase of c-Ret mRNA, the UB branching was reduced. Prior data observed in our Lab, showed a reduction of 28.3% in ureteric bud branches after 14.5 days of gestational protein restriction (Mesquita et al., 2010b) could be associate with a 28% reduction in labeling for Six2 cells in the MM, despite unchanged GDNF transcription.

 7. As a follow-up to my previous major comment 4, the authors state that they are currently performing studies to investigate autophagy and apoptosis pathways in nephron progenitor cells of metanephric mesenchyme during renal development, in the offspring of mice submitted to gestational protein restriction. Please include such data, at least part of them, in the current manuscript.

**R.: 7 comment**

Studies are being done in a doctorate thesis in our Lab using mice from the C57BL / 6-TgCAG-RFP / EGFP / Map1lc3b1Hill / J strain, transgenic autophagy. However, the studies are ongoing, and only preliminary results have been obtained.

 8. The authors have addressed my previous major comments 7, 11, and 12, however there are significant English problems in the corresponding texts.

**R.: 8 comment**

The grammatical and typographical errors have been edited.

 9. My previous major comment 10 was not properly addressed, since the authors’ statement “the current study established that the let-7 family of miRNAs promotes MYC expression through transcriptionally induced let-7 repressor, LIN28 enhancement and posttranscriptional expressed LIN28 RNA binding-protein, promoting downregulation upon LP kidney cells differentiation” would require generation of data on Lin28 expression.

**R.: 9 comment**

The question raised by the reviewer is pertinent. However, we do not have enough tissue from all the animals studied to perform the analyzes. In this way, we try not to be affirmative in the discussion of the work, just carefully suggesting the possibility that, at least in part, the suppressive effects of the expression of let-7 miRNAs are indirectly due to a reduction in lin28. As we answered above, we are carrying out complementary studies on the routes shown here, so we hope in the short term to have an answer to this question.

Thus, include the text below to read:

On the other hand, Yermalovich et al. (2019) have demonstrated that the overexpression of Lin28b, an RNA-binding protein, is associated with suppressive let-7 miRNAs. While the lin28 and let-7 genes are well-established regulators of ontogenic timing in invertebrates, the role of these in mammalian organ development was not fully understood. In the current study, the enhanced let-7a-5p miRNA expression in the LP fetus could be associated with reducing CM cell proliferation, compromising the whole nephrogenesis relative to the NP group. In this way, we may hypothesize the unprecedented findings of CM cell proliferation suppression and early cessation of nephrogenesis caused by increased let-7 miRNAs may occur directly or indirectly via the transient reduced expression of Lin28b in the 17-DG LP.
