# Supplementary material for: Impact of gestational low-protein intake on embryonic kidney microRNA expression and in nephron progenitor cells of the male fetus
Source: PLoS One. 2021 Feb 5;16(2):e0246289. doi: 10.1371/journal.pone.0246289 (PMC7864410; doi:10.1371/journal.pone.0246289)
Supplement: Attachment — Submitted filename: 9Response to Reviewers v.27-12-20.docx [file pone.0246289.s004.docx]

Response to Reviewers

PONE-D-20-15837

 The gestational low-protein intake impact in microRNA expression of the kidney progenitor cells in male offspring fetuses

Dear Editor:

We are attaching the Edited version and response to the Reviewers of the Manuscript PONE-D-20-15837. The gestational low-protein intake impact in microRNA expression of the kidney progenitor cells in male offspring fetuses submitted by Sene et al. resubmitted as a full paper. Practically, all manuscript sections were rewritten entirely, and many reviewer suggestions were now included in that new version. The grammatical and typographical errors have been corrected, and the manuscript was submitted to the editing service to review. The Introduction, Material, and Method and Discussion sections of the document were revised and completely rewritten to include reviewers' suggestions and comments.

Hopefully, this edited manuscript version could now be better considered for publication by this prestigious Journal.

Sincerely yours,

**Patricia Aline Boer, Ph.D**

**Internal Medicine Department, School of Medicine,**

**Campinas State University, Campinas, SP, Brazil.**

Reviewer 2 comments to the Author

Reviewer #2: My comments have been adequately addressed or justified, however two points remain to be fulfilled/considered.

1. The language quality gave one more step ahead, however it still needs improvement to reach an appropriate level. Incomplete sentences, inappropriately structured sentences and grammatical errors are still frequent, particularly in the discussion. At this point, therefore, my recommendation is to have an editing service review the manuscript.

Response from Authors

R.1: Practically, all manuscript sections were rewritten entirely, and many reviewer suggestions were now included in that new version. The grammatical and typographical errors have been corrected, and the manuscript was submitted to the editing service to review. The Introduction, Material, and Method and Discussion sections of the document were revised and completely rewritten to include prior reviewers' suggestions and comments.

2. The authors are presently performing studies to investigate autophagy and apoptosis pathways in nephron progenitor cells of metanephric mesenchyme during renal development in the offspring of mice submitted to gestational protein restriction. Initial data have been generated. Given the relevance of this issue to the present study and the additional time from the last revision, do the current results include any significantly robust piece of data that could expand this aspect in the present manuscript?

R2: Dear reviewer, the authors choose to present the results on autophagy and apoptosis in a future manuscript for two main reasons: 1. it is part of a doctoral study by another student, which includes a series of other evaluations, which at this moment only presents preliminary results; 2. Studies on this topic have suffered a significant delay since the University suspended on-site activities, practically throughout the year 2020, which caused a considerable delay in obtaining the results. This delay also includes the breeding and genetic control of mice from the C57BL / 6-TgCAG-RFP / EGFP / Map1lc3b1Hill / J strain, essential for reasonable experimental control. However, the studies are ongoing, and preliminary results have been obtained.
